# Effect of Blue Light on Endogenous Isopentenyladenine and Endoreduplication during Photomorphogenesis and De-Etiolation of Tomato (*Solanum lycopersicum* L.) Seedlings

**DOI:** 10.1371/journal.pone.0045255

**Published:** 2012-09-25

**Authors:** Véronique Bergougnoux, David Zalabák, Michaela Jandová, Ondřej Novák, Anika Wiese-Klinkenberg, Martin Fellner

**Affiliations:** 1 Laboratory of Growth Regulators, Faculty of Science, Palacky University and Institute of Experimental Botany A.S ČR v.v.i., Olomouc, Czech Republic; 2 Centre of the Region Haná for Biotechnological and Agricultural Research, Department of Molecular Biology, Faculty of Science, Palacky University, Olomouc, Czech Republic; 3 Department of Botany, Faculty of Science, Palacky University in Olomouc, Olomouc, Czech Republic; 4 Institute of Bio- and Geosciences, IBG-2: Plant Sciences, Forschungszentrum Jülich GmbH, Jülich, Germany; Instituto de Biología Molecular y Celular de Plantas, Spain

## Abstract

Light is one of the most important factor influencing plant growth and development all through their life cycle. One of the well-known light-regulated processes is de-etiolation, i.e. the switch from skotomorphogenesis to photomorphogenesis. The hormones cytokinins (CKs) play an important role during the establishment of photomorphogenesis as exogenous CKs induced photomorphogenesis of dark-grown seedlings. Most of the studies are conducted on the plant model *Arabidopsis*, but no or few information are available for important crop species, such as tomato (*Solanum lycopersicum* L.). In our study, we analyzed for the first time the endogenous CKs content in tomato hypocotyls during skotomorphogenesis, photomorphogenesis and de-etiolation. For this purpose, two tomato genotypes were used: cv. Rutgers (wild-type; WT) and its corresponding mutant (*7B-1*) affected in its responses to blue light (BL). Using physiological and molecular approaches, we identified that the skotomorphogenesis is characterized by an endoreduplication-mediated cell expansion, which is inhibited upon BL exposure as seen by the accumulation of trancripts encoding CycD3, key regulators of the cell cycle. Our study showed for the first time that iP (isopentenyladenine) is the CK accumulated in the tomato hypocotyl upon BL exposure, suggesting its specific role in photomorphogenesis. This result was supported by physiological experiments and gene expression data. We propose a common model to explain the role and the relationship between CKs, namely iP, and endoreduplication during de-etiolation and photomorphogenesis.

## Introduction

Light is one of the most important environmental factors that regulate plant behavior. This control by light is exerted as soon as a new plant starts to develop from a seed. In dicotyledonous plants, the hypocotyl connects the two cotyledons to the radicle. When a seed germinates in darkness, a so-called etiolated seedling develops, characterized by a fast growth rate of hypocotyl, closed cotyledons and a hook at the upper part of the hypocotyl, protecting the apical shoot meristem from any damage during the growth through the soil. As soon as the seedling emerges from the soil, it perceives light and switches to photomorphogenesis, characterized by a reduced growth rate of the hypocotyl, the opening and greening of cotyledons and the differentiation of etioplasts into chloroplasts, ensuring the autotrophy of the developing young plant. This process is referred as de-etiolation [1]. Plants have evolved highly sophisticated unique photoreceptors to perceive and respond to light. If one considers only the visible and near-infrared region of the light spectrum, three major classes of photoreceptor can be described: phytochromes (PHYs) that sense mainly the red/far-red part of the light spectrum and cryptochromes (CRYs) and phototropins (PHOTs) perceiving the blue part of the light. Although both red light (RL) and blue light (BL) induce de-etiolation, BL inhibits growth quicker and to a greater extent than RL [2]. Whereas the mechanisms of light perception are more and more understood, downstream events still remain to be elucidated. Nevertheless, it is now assumed that plant hormones play an important role in the light signaling pathway [3].

The cytokinins (CKs) were discovered more than 50 years ago due to their role in promoting cell division in tobacco tissue culture [4]. They are N^6^-substituted adenine derivatives, involved in numerous processes during plant growth and development, such as germination, leaf senescence, nodulation, and circadian rhythms. They also mediate light-regulated de-etiolation [5]–[6]. Cytokinin synthesis and catabolism are finely spatially and temporally regulated in order to ensure an appropriate homeostasis. Three main CKs are present in plants: isopentenyladenine (iP), *trans*-zeatin (*t*Z) and *cis*-zeatin (*c*Z). The role of CKs in photomorphogenesis was first reported in *Arabidopsis* [7]. Indeed, exogenously applied CKs were able to mimic the de-etiolation in seedlings grown in the dark. Concomitantly, the *Arabidopis amp1* mutant, accumulating a high concentration of CKs both in light and dark conditions, presented several characteristics of de-etiolation [8]. A model had been proposed in which light and CKs could act independently or sequentially to control downstream events of the light-regulated responses [9]. The convergence of the two signaling pathways was recently further described in *Arabidopsis* [10]. In fact, both CRY and CK signaling pathways were shown to induce the accumulation and stability of the HY5 (ELONGATED HYPOCOTYL5) protein, a key transcription factor mediating the photomorphogenesis.

As almost all cells of a hypocotyl are formed during embryogenesis, only a few cell divisions, restricted to the development of stomata, occur during etiolation [11]. In *Arabidopsis,* the hypocotyl is made of 20 epidermal cells and the cells elongate more than 100-fold of their embryonic length during etiolation mainly due to cell expansion [12]. Two processes govern the cell expansion: the increase in the cell ploidy by endoreduplication and the cell expansion itself, driven by water uptake [13]. During the endoreduplication, characterized by a repetitive chromosal DNA synthesis without mitosis [11], [14], the cell cycle oscillates between the G1/S phases and does not undergo the G2/M transition. Cyclin-dependent kinases (CDKs) and their interacting partners, cyclins (CYCs), are key regulators of the cell cycle. The cyclin family is very complex, counting 49 different genes in *Arabidopsis* and organized into seven different subclasses (A, B, C, D, H, P and T). D-type cyclins (CYCD) drive cells into the G1/S transition but also probably into the G2/M transition [15]. In *Arabidopsis*, the CYCDs are organized into seven subgroups, among which the D3-type cyclins are of particular interest in endoreduplication. Indeed it was demonstrated that these cyclins both maintain cells in mitotic cycle and restrain the transition to endocycling [16]. Recent studies report a relationship between CKs and endoreduplication. Indeed, the expression of *CYCD3* genes was stimulated in *Arabidopsis* mutants with a high content of CKs or by exogenous application of CKs [17]. More recently, the role of CYCD3 in mediating responses to CK was described [16].

The present study focused on investigating the status of endogenous CK and endoreduplication during photomorphogenesis and de-etiolation of tomato seedlings under exposure to BL. We identified a raise in iP content under BL condition correlating with the inhibition of cell expansion. The use of exogenous CKs supported our hypothesis. We also identified that BL induced the inhibition of endoreduplication probably by the mean of the CYCD3. The relationship between endoreduplication and CKs (iP) during the BL-mediated photomorphogenesis and de-etiolation of tomato is discussed.

## Results

### Characterization of the Early Development of *7B-1* Mutant Seedlings Grown in Continuous BL

**Figure 1:**
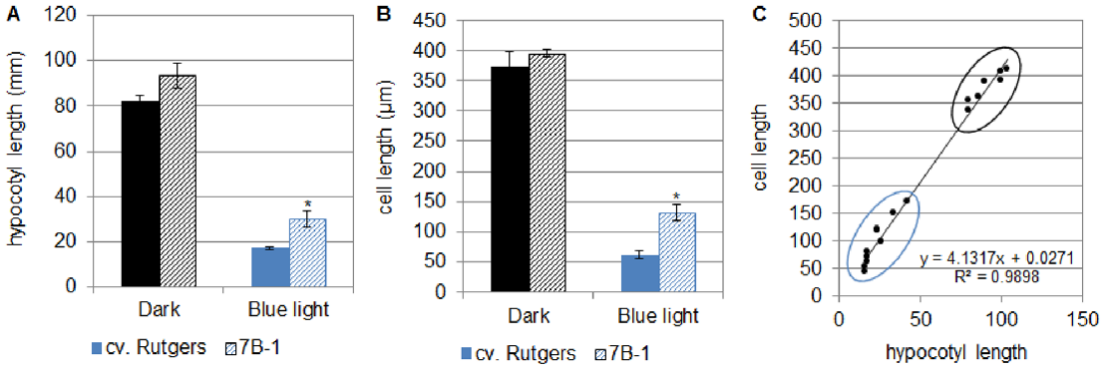
Length of the hypocotyl (A), length of the epidermal cell of hypocotyl (B) of cv. Rutgers and the *7B-1* mutant, and correlation between hypocotyl length and epidermal cell length (C). Seedlings were grown for 5 days in the D or in continuous BL (10 µmol.m^−2^.s^−1^). Results represent the average ± SE (n = 10 for hypocotyl). *significantly different from cv. Rutgers (two-way ANOVA, Bonferroni *post-hoc* test, p<0.05.

The *7B-1* mutant of tomato was characterized in the early 90′s based on its photoperiod-dependent male sterility [18]. Several reports demonstrated that the mutant was less affected in several responses to BL [19]–[21]. In this study, the growth of the mutant under BL was investigated. For this purpose, seedlings of both genotypes (cv. Rutgers-WT and mutant) were grown for 5 days either in the D or in continuous BL before measuring the length of the hypocotyl (Figure 1A). When the mutant was grown in the D, no significant difference in the hypocotyl length could be observed compared to WT. When grown under continuous BL, the growth of both genotypes was strongly and significantly reduced; nevertheless the mutant showed significantly longer hypocotyls than the WT (+74%).

The longer hypocotyl of the *7B-1* mutant grown in continuous BL can result from either higher cell division rate or higher cell expansion compared to the WT. In order to answer this question, the length of epidermal cells was measured in the hypocotyl of both genotypes grown for 5 days either in the D or in continuous BL (Figure 1B). In the D-grown seedlings, no significant difference was observed between the two genotypes. When seedlings were grown in continuous BL, the length of hypocotyl epidermal cells was significantly reduced compared to D-grown seedlings. Nevertheless, the epidermis of the mutant hypocotyl was constituted of significantly longer cells compared to the WT. Taken together, these results first demonstrated a strong correlation between hypocotyl length and epidermal cell length (Figure 1C; Pearson’s coefficient: r = 0.98, p<0.05), then that the BL-induced inhibition of growth is characterized by the inhibition of the expansion of the hypocotyl epidermal cell and finally that the mutant is affected in this physiological process.

### Endoreduplication in the *7B-1* Mutant Seedlings Grown in Continuous BL

**Figure 2:**
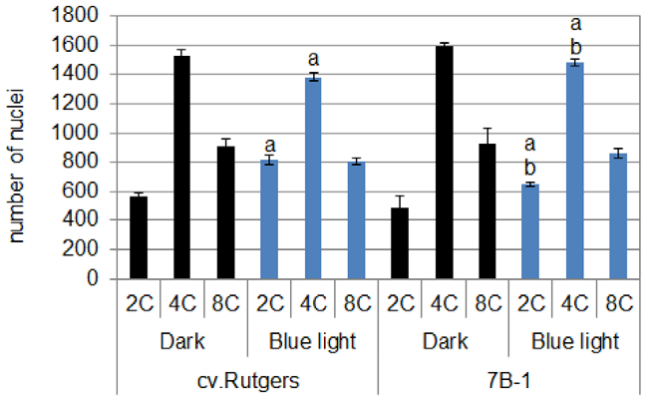
Analysis of the ploidy of the epidermal cells of the hypocotyl of cv. Rutgers and the *7B-1* mutant after 5 days of growth in the D or in continuous BL (10 µmol.m ^−**2**^ **.s** ^−**1**^ **).** Results represent averages ± SE (n = 10). a: significantly different from the D condition; b: significantly different from cv. Rutgers grown in the same condition (two-way ANOVA, Bonferroni *post-hoc* test, p<0.05).

Two processes govern the cell expansion: the increase in the cell ploidy by endoreduplication and the cell expansion itself [13]. The ploidy of the hypocotyl epidermal cell was analyzed by flow cytometry for the two genotypes grown for 5 days in the D or continuous BL (Figure 2). In both genotypes, three levels of ploidy could be determined: 2C, 4C and 8C. In the WT, the 4C nuclei were the most abundant among the nuclei detected. When sedlings were grown in the D, no significant difference could be observed between the two genotypes. The growth in continuous BL was characterized by an increase in the number of 2C nuclei and a decrease in 4C nuclei in both genotypes. Nevertheless, in the *7B-1* mutant, the number of 2C nuclei and 4C nuclei were respectively less and more abundant than in the WT.

**Figure 3:**
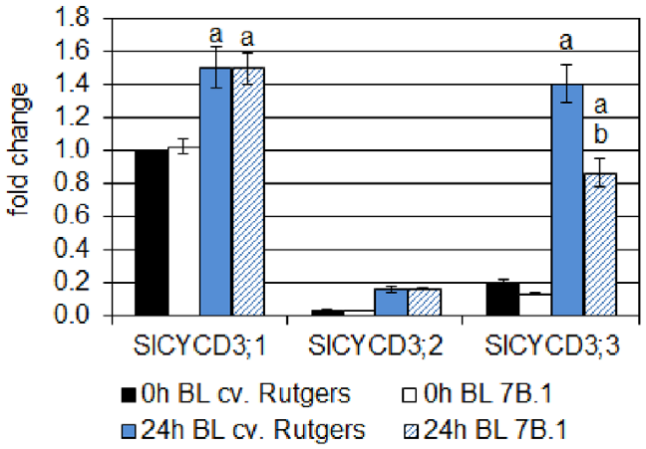
Analysis by qRT-PCR of the expression of three *CycD3* genes of tomato in the hypocotyl of cv. Rutgers and *7B-1* seedlings grown for 2 days in the D (0 **h BL) and transferred for 24** **h in continuous BL (10 µmol.m** ^−**2**^ **.s** ^−**1**^ **; 24** **h BL).** Results represent averages ± SE of three independent biological repeat. The *EF1α* gene was taken as housekeeping gene. The relative quantification was made against the expression of *SlCYCD3;1* from the sample ‘0 h BL cv. Rutgers’. Statistics were done separately for each gene; a: significantly different from the D condition; b: significantly different from cv. Rutgers in the same light condition (two-way ANOVA, Bonferroni *post-hoc* test, p<0.05).

A close correlation between the expression of *CycD3* genes, key regulators of the cell cycle and endoreduplication, and the ploidy level of the cells of different organs was previously described [16]. In tomato, three *CycD3* genes have been characterized: *CycD3;1* (accession number: AJ002588), *CycD3;2* (accession number: AJ002589) and *CycD3;3* (accession number: AJ00259) [22]. Their expression was investigated by qRT-PCR in the upper part of the hypocotyl of 2-day old (do) etiolated seedlings and in 2 do-etiolated seedlings further subjected for 24 h to continuous BL (Figure 3). The expression of *SlCycD3;2* was very weak compared to the two other *CycD3* and no significant difference in its expression was observed between genotypes independently from the light quality. The *SlCycD3;1* transcripts were the most abundant in the hypocotyl of the etiolated seedlings of both genotypes. The exposure for 24 h to BL of the etiolated seedlings induced a significant accumulation of this transcript in the hypocotyl of both genotypes (+50%). By contrast, the *SlCycD3;3* transcripts, low abundant in the hypocotyl of etiolated seedlings of both genotypes, were drastically accumulated in the WT (7 times higher compared to the D) after exposure to BL; this accumulation was not so important in the hypocotyl of the *7B-1* mutant (4 time higher compared to the D). An extended growth for 24 h in the D induced an inhibition of the expression of the 2 genes, confirming that the increase in the expression of the 2 genes was specific of the response to the BL exposure (Figure S1).

### Accumulation of CKs in the Hypocotyl of Young Tomato Seedlings Grown in BL

**Figure 4:**
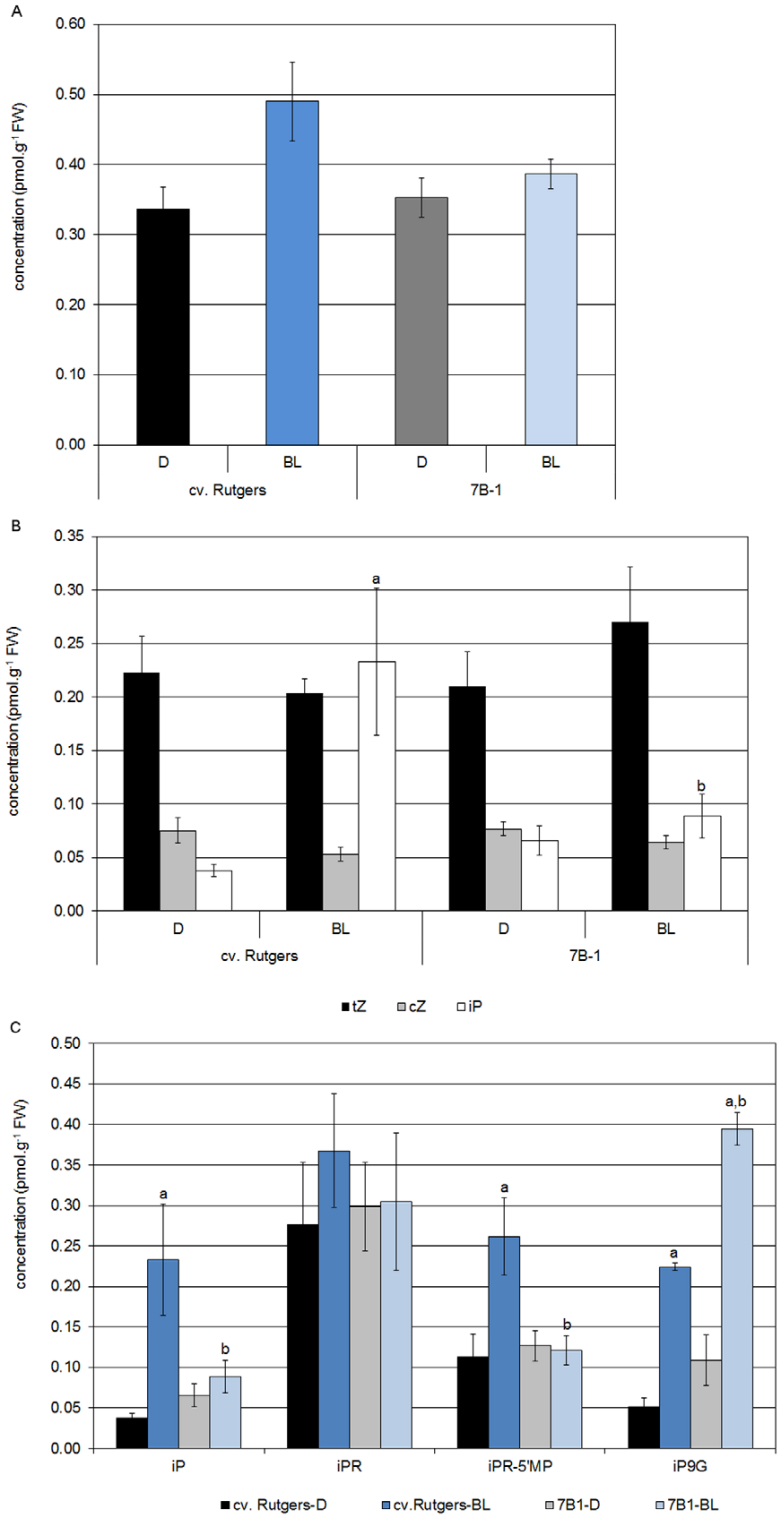
Analysis of the total free-bases cytokinin (A), of individual free-bases cytokinins (B), and of iP-derivatives (C) in the hypocotyl of seedlings of cv. Rutgers and the *7B-1* mutant after 5 days of growth in the D or continuous BL (10 µmol.m ^−**2**^ **.s** ^−**1**^ **).** Results represent averages ± SE of 4 independent biological repeats. Statistics were done separately for each compound; a: significantly different from the D condition; b: significantly different from cv. Rutgers in the same light condition (two-way ANOVA, Bonferroni *post-hoc* test, p<0.05).

The content of total free-bases CKs was quantified in the hypocotyls of the WT and the *7B-1* mutant grown for 5 days in the D or in continuous BL (Figure 4A). The total free-base CK content of hypocotyl of D-grown seedlings did not differ between the two genotypes. BL induced the accumulation of total free-base CKs significantly more in the WT than in the *7B-1* mutant. The content of iP, *t*Z and *c*Z was further specifically determined in order to understand the particular role of each CK (Figure 4B). The *c*Z content was very low in the hypocotyls of both genotypes and no difference was observed whatever the light conditions tested. In contrast, the *t*Z was the most abundant CK present in etiolated seedlings of both genotypes but no significant difference could be observed between either genotypes or light condition. The results obtained from iP quantification were the most interesting. Indeed, the iP content was the same in the hypocotyls of the two D-grown genotypes. When seedlings were grown in continuous BL, only the WT strongly and significantly accumulated iP. In the *7B-1* mutant, the iP content was identical in D- and BL-grown hypocotyl (Figure 4B). This differential accumulation of iP between the two genotypes was associated with a significant stimulation of the synthesis in the WT, as shown by the accumulation of the riboside 5′-monophosphate precursor, and a stimulation of the inactivation of the free base in the *7B-1* mutant, as shown by the accumulation of the 9-glucoside form of iP in the hypocotyl of the *7B-1* mutant grown in continuous BL (Figure 4C).

### Effect of Exogenous CKs and Inhibitor of CK Receptors on the Growth of Tomato Seedlings

These last results led us to the conclusion that iP could be part of the mechanisms involved in the BL-induced inhibition of growth. In order to verify this hypothesis the capacity of exogenously applied iP, *t*Z and *c*Z to inhibit the growth of the *7B-1* mutant seedlings was investigated. Indeed, one could expect that exogenous iP will overcome the endogenous iP deficiency observed in the mutant, further inhibiting the growth of the mutant when grown in the BL. For this purpose, 2-do etiolated seedlings of the *7B-1* mutant were transferred under green safety light on medium containing different concentration of iP, *t*Z or *c*Z. Dishes were placed in continuous BL for 24 h before the length of the hypocotyl was measured. The iP was found to inhibit the growth of the *7B-1* mutant in a concentration-dependent manner, whereas *t*Z and *c*Z seemed to have no effect on the growth of the mutant (Table 1).This experiment led us to the conclusion that iP plays an important role during BL-induced inhibition of growth.

**Table 1:** Effect of exogenous CK on the growth of *7B-1* mutant seedlings grown in continuous BL (10 µmol.m^−2^.s^−1^).

		iP			tZ				cZ		
	control	1 µM	5 µM	10 µM		1 µM	5 µM	10 µM	1 µM	5 µM	10 µM
Length of hypocotyl (mm)	51.4±1.3	47.5±1.5	45.5±1.5	43.4±1.5^a^		50.1±1.4	50.2±1.5	50.3±1.3	50.5±1.4	50.3±1.4	50.4±1.4
Inhibition of growth (%)		−7.0%	−10.9%	−15.0%		−0.1%	−1.6%	−1.5%	−1.2%	−1.4%	−1.3%

Two day-old etiolated seedlings were transferred on MS medium containing different concentration of iP, tZ or cZ and grew for 24 h in continuous BL. Results represent averages ± SE of 3 independent biological repeat with n = 10 to 15. a: significantly different from other condition (two-way ANOVA, Bonferroni post-hoc test, p<0.05).

**Figure 5:**
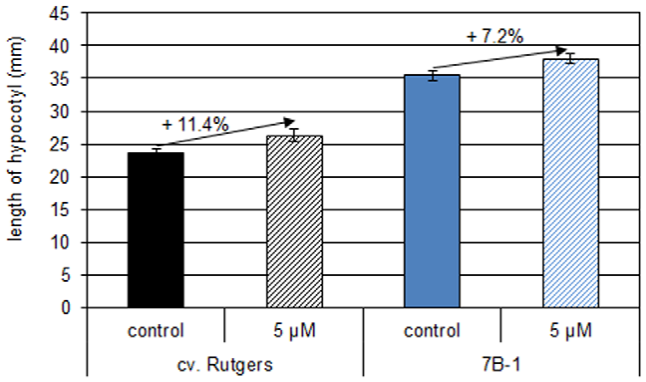
Effect of LRG-911, an inhibitor of CK receptor, on the growth of WT and *7B-1* mutant seedlings grown in continuous BL (10 µmol.m ^−**2**^ **.s** ^−**1**^ **).** Two day-old etiolated seedlings were transferred on MS medium containing 5 µM LRG-911 and grew for 24 h in continuous BL. Results represent averages ± SE (n = 30). *significantly different from the control (*t*-test of student, p<0.05).

The effect of LRG-911, an inhibitor of CK receptors, was investigated. For this purpose, 2-do etiolated seedlings of both genotypes were transferred under green safety light on medium containing 5 µM LRG-911. Dishes were placed in continuous BL for 24 h before the length of the hypocotyl was measured (Figure 5). The addition of LRG-911 in the medium induced a significant stimulation of the growth of the hypocotyl of both genotypes when seedlings were grown in continuous BL. Nevertheless, LRG-911 was less efficient to stimulate the growth of the *7B-1* mutant than of the WT.

### Regulation of *SlLOG* Genes by BL

**Figure 6:**
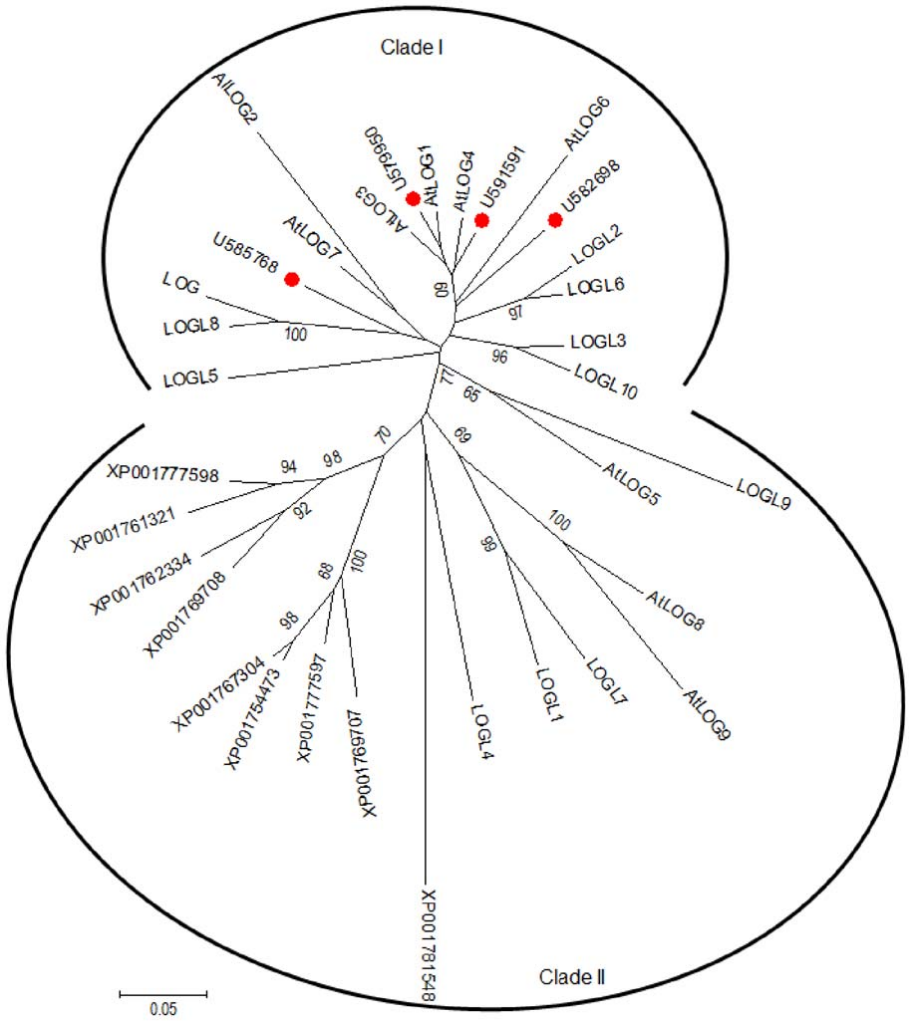
Unrooted phylogenetic tree of LOG and homologs in tomato (red dots), *Arabidopsis* (AtLOGs), rice (LOG and LOGLs) and moss (XP). For *Arabidopsis*, rice and moss, sequences used were those described in Kuroha *et al.* (2009) [24]. For tomato, sequences were obtained from the SOL genomic resource after BLAST with *AtLOG1*. The phylogenetic tree was constructed in MEGA5.05 program (hppt://www.megasoftware.net) using the neighbor-joining method with 1000 bootstrapping replicates. The bootstrap values above 60% are indicated on the tree. The scale represents 0.05 amino acid substitutions per site.

The free-base CKs are directly produced from the corresponding cytokinin riboside 5′-monophosphate by the activity of the protein encoded by *LOG* (*LONELY GUY*) genes [23]. In order to better understand the regulation of CK metabolism during BL-induced de-etiolation, orthologs of *Arabidopsis LOG* genes were identified in tomato from a Blast analysis in the SOL Genomics Network database (http://solgenomics.net/) using *AtLOG1* sequence as a query. Four full-length sequences were identified and annotated according their identity to *Arabidopsis* sequences: *SlLOG1* (SGN-U579950), *SlLOG4* (SGN-U591591), *SlLOG2* (SGN-U585768) and *SlLOG6* (SGN-U582698). The deduced SlLOG proteins are composed of 218 (SlLOG1 and SlLOG4), 226 (SlLOG2) and 234 (SlLOG6) amino acids. Tomato LOG sequences were introduced into the phylogenetic tree built by Kuroha and coworkers, comprised of the *LOG*-like genes of *Arabidopsis*, moss and rice [24]. The introduction of the tomato LOGs did not change the organization of the tree into two clades. The sequences identified from tomato belonged to the clade I (Figure 6).

**Figure 7:**
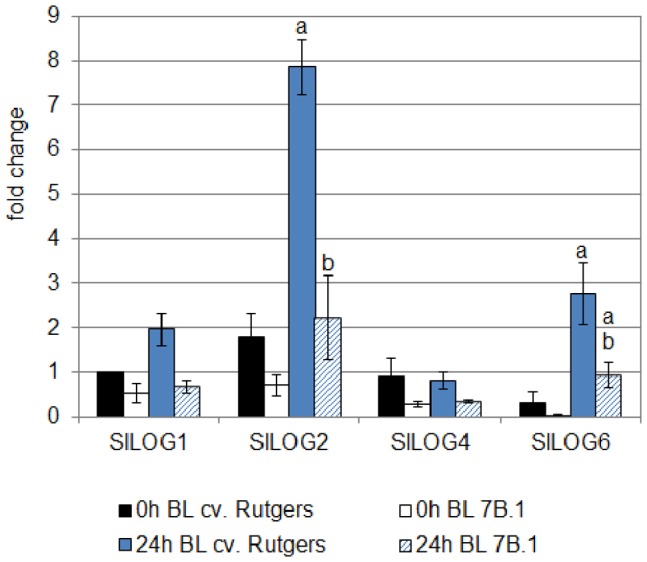
Analysis by qRT-PCR of the expression of four *LOG* genes of tomato in the hypocotyl of cv. Rutgers and *7B-1* seedlings grown for 2 days in the D (0 **h BL) and transferred for 24** **h in continuous BL (10 µmol.m** ^−**2**^ **.s** ^−**1**^ **; 24** **h BL).** Results represent averages ± SE of three independent biological repeat. The *EF1α* gene was taken as housekeeping gene. The relative quantification was made against the expression of *SlLOG1* from the sample ‘0 h BL cv. Rutgers’. a: significantly different from the D condition; b: significantly different from cv. Rutgers in the same light condition (One-way ANOVA, Bonferroni *post*-*hoc* test: p<0.05).

The expression of the four *SlLOG* genes was investigated by qRT-PCR in the upper part of the hypocotyl of both genotypes (Figure 7). In the case of the WT, except for *SlLOG4*, the exposure to BL induced the accumulation of *LOG* transcripts, with *SlLOG2* being the most accumulated. In the *7B-1* mutant, the BL-induced accumulation of the different transcripts was not found to be so significant, except for *SlLOG6*. Nevertheless, this transcript was still less abundant in the mutant than in the WT. These results show a nice correlation between the BL-induced accumulation of the genes involved in CK synthesis and the accumulation of iP under BL condition. Moreover as the *SlLOG2* was the most abundant among the four transcripts it could be hypothesized that it is the most probably responsible for the observed synthesis of iP. An extended growth for 24 h in the D did not affect the expression of *SlLOG1*, *SlLOG2* and *SlLOG 6* and even inhibited the expression of *SlLOG4*, confirming that the changes in expression were specific of the response to the exposure to BL (Figure S2).

### Inhibition of CK Catabolism by BL

**Figure 8:**
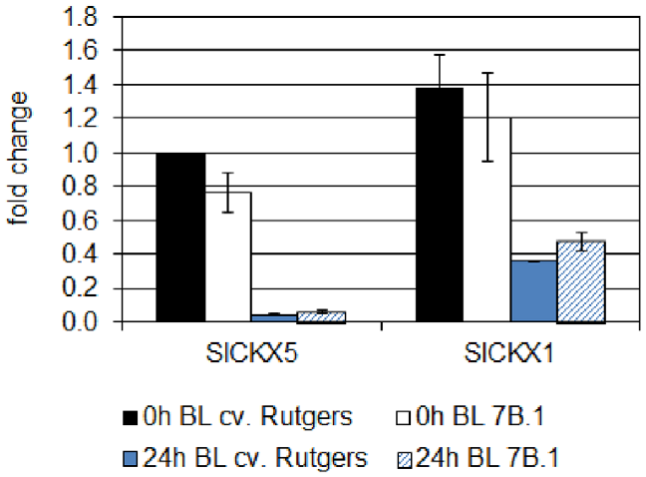
Analysis by qRT-PCR of the expression of two *CKX* genes of tomato in the hypocotyl of cv. Rutgers and *7B-1* seedlings grown for 2 days in the D (0 **h BL) and transferred for 24** **h in continuous BL (10 µmol.m** ^−**2**^ **.s** ^−**1**^ **; 24** **h BL).** Results represent averages ± SE of three independent biological repeat. The EF1α gene was taken as housekeeping gene. The relative quantification was made against the expression of *SlLOG1* from the sample ‘0 h BL cv. Rutgers’. A two-way ANOVA with Bonferroni post-test was performed, taking separately each gene; no significant difference was observed.

The homeostasis of CKs is a complex phenomenon based on synthesis, import rate, association and dissociation from conjugates (especially glucosides), export and catabolism [25]–[26]. Enzymes responsible for the irreversible degradation of CKs are cytokinin dehydrogenases (CKXs), which cleave the active CK into adenine and the corresponding aldehyde [27]. Three ESTs were identified from the SOL Genomics Network database and were annotated based on their homology with *Arabidopsis* CKXs: SGN-U342971/*SlCKX1*; SGN-U336412/*SlCKX3* and SGN-U223622/*SlCKX5* (H. Pospíšilová, unpublished data). Using qRT-PCR, the expression of the three *SlCKX* genes was analyzed in the upper part of the hypocotyl of both genotypes (Figure 8).

The expression of *SlCKX3* was barely detectable, suggesting that this gene is unlikely to be involved in the process (data not shown). No significant difference in the accumulation of the *SlCKX1* and *SlCKX5* transcripts in the hypocotyl of etiolated seedlings was observed between the two genotypes. The exposure to BL induced in both genotypes the inhibition of the accumulation of the two transcripts. Whereas *SlCKX5* was the most repressed by BL, no significant difference could be observed again between the two genotypes. An extended growth for 24 h in the D stimulated the expression of the 2 genes, confirming that the inhibition of their expression was specific of the response to the BL exposure (Figure S3).

### Characterization of the BL-induced De-etiolation of Tomato Seedlings

**Figure 9:**
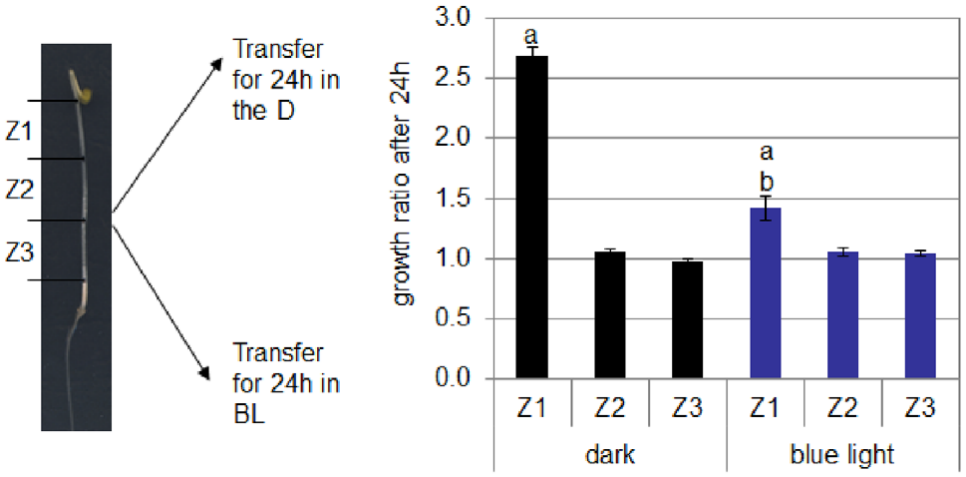
Growth of different zones of the hypocotyl of 2 day-old etiolated seedlings of cv. Rutgers in the D or in continuous BL (10 µmol.m ^−**2**^ **.s** ^−**1**^ **).** Data represent average ± SE (n = 8); a: significantly different from the other zones in the same light condition; b: significantly different from Z1 in the D (two-way ANOVA, Bonferroni *pos*t-*hoc*: p<0.05).

**Figure 10:**
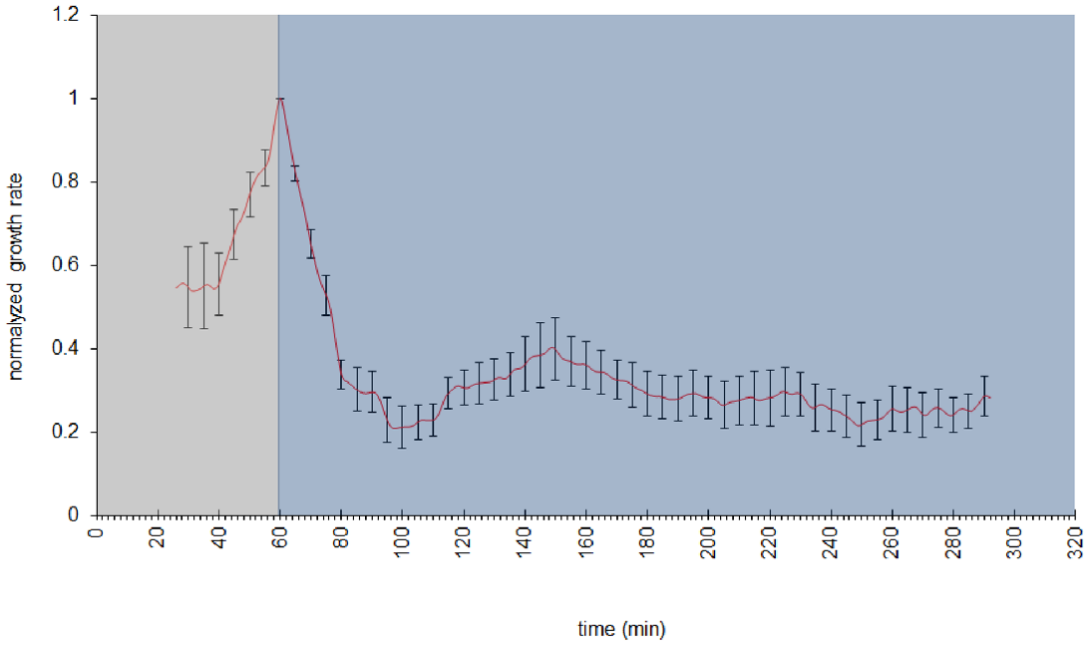
Evolution of the growth rate of the upper part of the hypocotyl of 2 day-old etiolated seedlings of cv. Rutgers during de-etiolation. Seedlings were first placed for 1 h in the D in order to equilibrate the seedling in its environment, BL (10 µmol.m^−2^.s^−1^) was then switched on and the growth rate was followed during 4 h. Data represent average ± SE (n = 11).

De-etiolation characterizes the switch from the skotomorphogenesis to the photomorphogenesis which normally occurs when a seed germinates into the soil and grows to give rise to a plantlet. In tomato, like in *Arabidopsis*, the D-induced expansion of the hypocotyl occurs along a basipetal gradient. The upper part of the hypocotyl, below the cotyledons, is the zone of active cell expansion and responds to the exposure to light (Figure 9). In order to gain more insights on the de-etiolation of tomato, the growth rate of the upper part of the hypocotyl was followed in 2-do etiolated seedlings exposed to BL (Figure 10). The exposure to BL induced a rapid and strong inhibition of the growth of the hypocotyl, the maximum of the growth rate inhibition being reached after 30–35 min of the onset of BL. A slight recovery of the growth rate followed and finally a steady low growth rate was established, corresponding to 30% of the dark growth rate.

### Cytokinins during BL-induced De-etiolation of Tomato Seedlings

**Figure 11:**
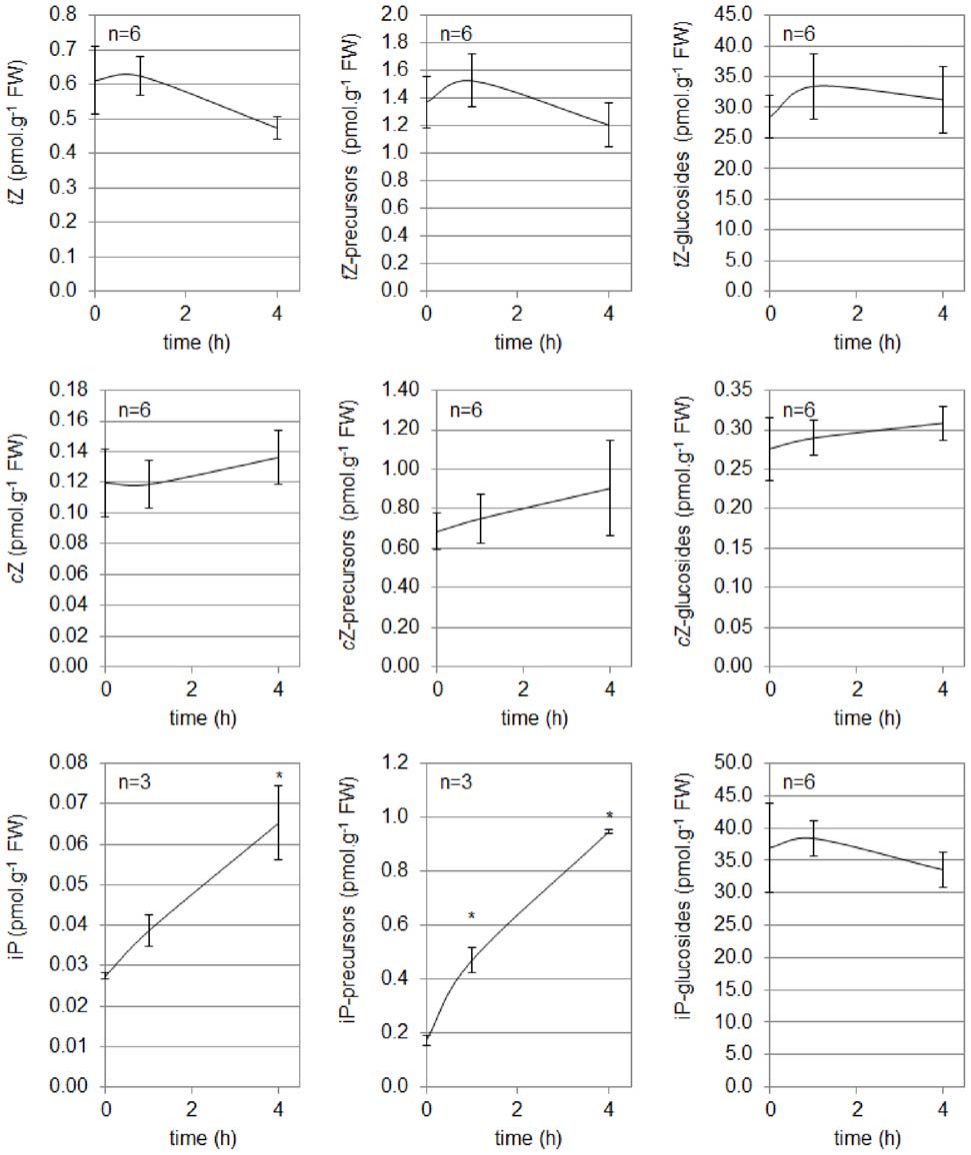
Analysis of the 3 main cytokinins (*t*Z, *c*Z and iP) and their derivatives in the upper part of the hypocotyl of cv. Rutgers grown for 2 days in the D and transferred in continuous BL (10 µmol.m ^−**2**^ **.s** ^−**1**^ **).** “Precursors” represent ribosides and monophosphate ribosides; “glucosides” correspond to the O-glucoside forms (-OG, -ROG, -7G and -9G). Results represent averages ± SE (n = number of biological repeats). *: significantly different from other conditions (one-way ANOVA, Bonferroni *post-hoc* test, p<0.05).

The content of *t*Z, *c*Z and iP and their derivatives was quantified in the upper part of the hypocotyls of 2-do etiolated WT seedlings after 1 h and 4 h of exposure to BL (Figure 11). No significant variation in *t*Z and *c*Z content could be observed during de-etiolation, even if it is tempting to assume a decrease in *t*Z and an increase of *c*Z. The exposure to BL of the etiolated seedlings did not affect the content in precursors and glucosides of these two types of CK. The most interesting results were observed for iP and derivatives. Indeed as soon as after 1 h of illumination, the content of free iP increased in the upper part of the etiolated hypocotyl and this increase was found to be significant after 4 h. The increase of free iP was correlated with a significant increase in iP-precursor content within 1 h of exposure to BL; the content of iP-glucosides was not found to be affected by the exposure to BL.

**Figure 12:**
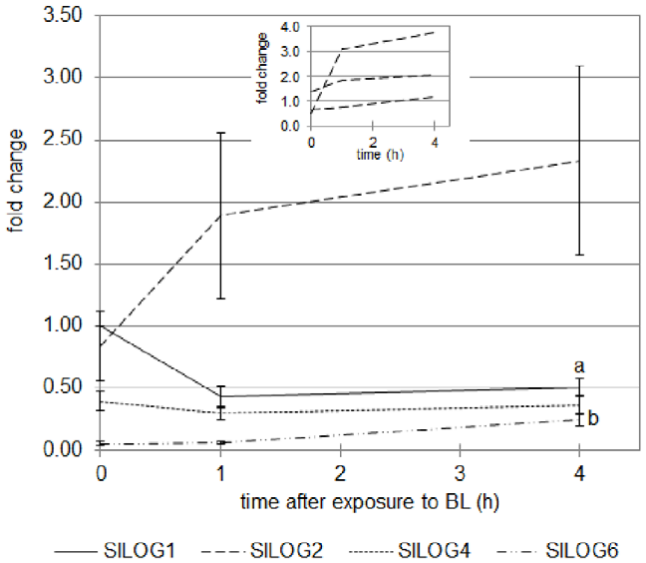
Analysis by qRT-PCR of the expression of four *LOG* genes of tomato in the upper part of the hypocotyl of cv. Rutgers seedlings grown for 2 days in the D then transferred to continuous BL (10 µmol.m ^−**2**^ **.s** ^−**1**^ **).** Results represent the averages ± SE of 3 independent biological repeats. The *EF1α* gene was taken as housekeeping gene. The relative expression was made against the expression of *SlLOG1* gene in the 2 day-old seedlings unexposed to BL. a: the expression of *SlLOG1* gene is significantly different from the expression in the 2 do seedlings unexposed to BL; b: the expression of *SlLOG6* gene is significantly different from the expression in the 2 do seedlings unexposed to BL (one-way ANOVA, Bonferroni post-hoc: p<0.05). The frame represents the data obtained for *SlLOG2* gene in the 3 independent biological repeats, showing the same trends despite the variability between repeats.

The expression of the four *SlLOG* genes was investigated by qRT-PCR in the upper part of the WT hypocotyl during de-etiolation (Figure 12). A differential regulation of the four *SlLOG* genes could be observed. Indeed, the expression of *SlLOG4* remained unaffected by the exposure to BL, while the expression of *SlLOG1* was significantly inhibited. The most interesting results were obtained for *SlLOG2* and *SlLOG6* genes whose expression was stimulated during the exposure to BL. While the results obtained for *SlLOG6* were found significant, those obtained for *SlLOG2* were not. This can be explained by the variability between the 3 biological repeats, leading to the hypothesis that the regulation of *SlLOG2* transcription is highly sensitive to light.

**Figure 13:**
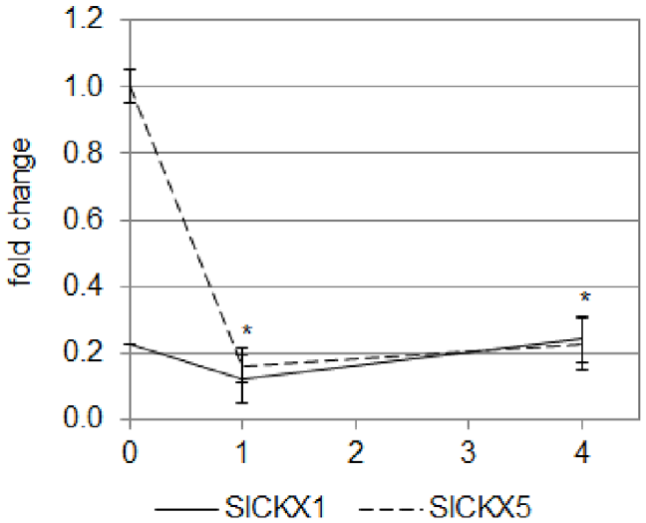
Analysis by qRT-PCR of the expression of two *CKX* genes of tomato in the upper part of the hypocotyl of cv. Rutgers seedlings grown for 2 days in the D then transferred to continuous BL (10 µmol.m ^−**2**^ **.s** ^−**1**^ **).** Results represent the averages ± SE of three independent biological repeats. The *EF1α* gene was taken as housekeeping gene. The relative expression was made against the expression of *SlCKX1* gene in the 2-do seedlings unexposed to BL. *: the expression of *SlCKX5* gene is significantly different from the expression in the 2-do seedlings unexposed to BL (one-way ANOVA, Bonferroni *pos*t-*hoc*: p<0.05).

The expression of *SlCKX1* and *5* was analyzed by qRT-PCR during the de-etiolation (Figure 13). Whereas the expression of *SlCKX1* was unaffected during the exposure to BL the expression of *SlCKX5* rapidly dropped within the first hour of the experiment.

### Endoreduplication during BL-induced De-etiolation of Tomato Seedlings

**Figure 14:**
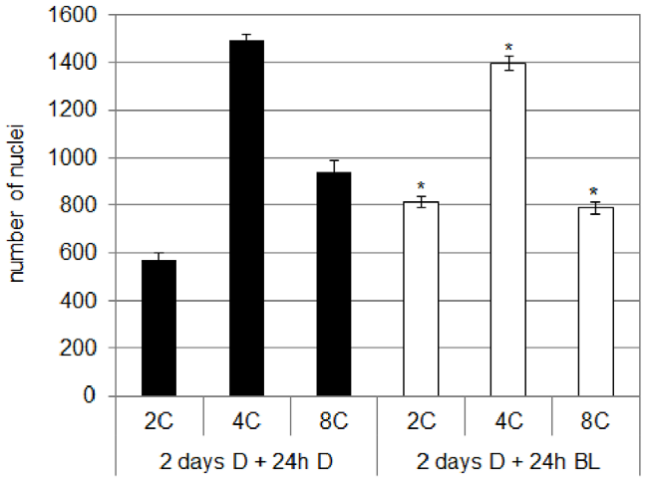
Analysis of the ploidy of the epidermal cell during de-etiolation of cv. Rutgers. Germinated seeds were grown for 2 days in the dark (D) in order to induce etiolation; etiolated seedlings were either kept in the dark (+24 h D) or transferred to continuous blue light (10 µmol.m^−2^.s^−1^; +24 h BL). *significantly different from the dark condition (two-way ANOVA, Bonferroni *post*-*hoc* test, p<0.05).

The ploidy of the epidermal cell was determined from the upper part of the hypocotyl of 2-do etiolated seedlings kept for 24 h either in the D or in continuous BL (Figure 14). In the D, the epidermis of the upper part of the hypocotyl is mainly constituted of 4C and 8C cells. When the seedlings are transferred to BL for 24 h, the proportion of 4C and 8C cells significantly decreased in favor to 2C cells.

**Figure 15:**
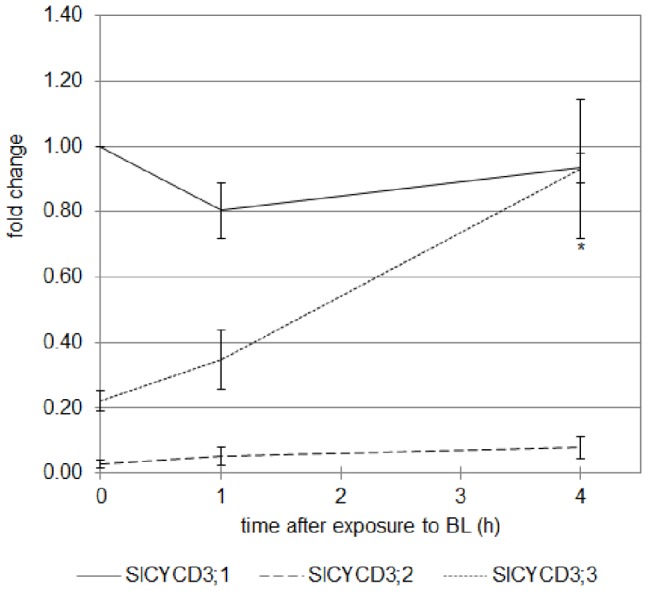
Analysis by qRT-PCR of the expression of the three *CycD3* genes of tomato in the upper part of the hypocotyl of cv. Rutgers seedlings grown for 2 days in the D then transferred to continuous BL (10 µmol.m ^−**2**^ **.s** ^−**1**^ **).** Results represent the averages ± SE of three independent biological repeats. The *EF1α* gene was taken as housekeeping gene. The relative expression was made against the expression of *SlCycD3;1* gene in the 2-do seedlings unexposed to BL. *: the expression of *SlCycD3;1* gene is significantly different from the expression in the 2-do seedlings unexposed to BL (one-way ANOVA, Bonferroni *pos*t-*hoc* test, p<0.05).

The expression of the three *SlCycD3* genes was investigated by qRT-PCR in the upper part of the hypocotyl of 2-do etiolated seedlings undergoing de-etiolation (Figure 15). The expression of *SlCycD3;2* was very weak compared to the two other *CycD3* genes and no significant difference in its expression was observed during de-etiolation. Despite the fact that *SlCycD3;1* was the most abundant in the hypocotyl of the etiolated seedlings, its expression remained unaffected by the exposure to BL. In opposite, the expression of *SlCycD3;2* was rapidly stimulated upon exposure to BL and the stimulation was found significant after 4 h.

## Discussion

Light is one of the most important environmental factor influencing the plant growth and development. One of the physiological responses under light control is de-etiolation. De-etiolation refers to the switch from the skotomorphogenesis (dark-stimulated growth) to the photomorphogenesis (light-controlled growth). Both red light (RL) and blue light (BL) stimulate de-etiolation by the mean of the RL-photoreceptors (phytochromes) and the BL-photoreceptors (cryptochromes and phototropins), respectively [28]. In addition to light, phytohormones play an important role in de-etiolation [29]. Most of the knowledge concerning de-etiolation and early event of photomorphogenesis is available from the plant model *Arabidopsis*, whereas little or no information is available from important crop species, such as tomato (*Solanum lycopersicum* L.). Mechanisms governing the hypocotyl growth differ in the dark from those in the light, even if the hypocotyl growth in the dark and in the light does not require the division of cortical or epidermal cells. Thus, for example, in the dark, cells elongate along an acropetal spatial and temporal gradient, whereas in the light all epidermal cells elongate continuously during the entire growth period [30]. In tomato, the epidermal cells of the hypocotyl developed also along an acropetal gradient in the seedlings grown in the dark with the zone situated under the cotyledons being the zone of active elongation. The use of continuous light regime inhibited the fast etiolated growth of the tomato hypocotyl with BL being more efficient than RL (data not shown), which was consistent with studies on different species [2]. In order to get more insights concerning skotomorphogenesis and photomorphogenesis in tomato, the spontaneous *7B-1* mutant was included in this study. The *7B-1* mutant, described for the first time in the early 90′s as a spontaneous photoperiod-dependent male sterile mutant [18], showed a reduced sensitivity to the BL-induced photomorphogenesis compared to the WT. This was in agreement with previous reports demonstrating that the mutant was less affected in several responses to BL [19]–[21]. Despite attempts to characterize it, the mutation remains unknown but several lines of evidence led to the conclusion that the *7B-1* mutant is not affected in cryptochrome 1 [20] and could be affected in phototropin signaling pathway (unpublished, personal data). In tomato, the length of the hypocotyl was tightly correlated with the length of the hypocotyl epidermal cells, i.e. longer the hypocotyl was and longer the epidermal cells were. Several lines of evidence indicate a correlation between the cell size and the DNA content [11]. The DNA content or ploidy is regulated by the process of endoreduplication which is characterized by a repetitive chromosomal DNA synthesis without mitosis or cytokinesis leading to an increase of nuclear DNA content [11], [14]. In the dark-grown hypocotyls of *Arabidopsis*, cells undergo an additional round of endoreduplication compared to the cells of the hypocotyl grown in the light, in close correlation with the size of the epidermal cells [30]. In tomato, we evidenced that the hypocotyl of seedlings grown in continuous blue light contained a higher proportion of 2C cells and a lower proportion of 4C cells than the hypocotyl of seedlings grown in the dark. The fact that the hypocotyl of the *7B-1* mutant contained less 2C cells and more 4C cells than the WT indicates that the endoreduplication is under the control of blue light. The role of the light in the control of endoreduplication was already mentioned. But if it had been evidenced a role of red/far-red light in the control of endoreduplication by the mean of phytochromes, the inhibition of endoreduplication by BL was not observed [31]. This last result is in contrast with the results presented here. One explanation could be that these authors determined the ploidy on the entire hypocotyl whereas our data focused on the analysis of the epidermal cells of the hypocotyl. This is further supported by the fact that no difference in ploidy level could be observed when the entire hypocotyl of tomato was used in experiments (data not shown). It is supposed that endoreduplication results from a modification of the mitotic cycle [32]. Cyclin-dependent kinases (CDKs) and their interacting partners, cyclins (CYCs), are key regulators of the cell cycle. Among the vast family of CYCs, the D-type cyclins (CYCD) were found to be associated with the endoreduplication. Thus, in *Arabidopsis*, CYCD3 loss-of-function mutants were not affected in the size of their leaves or petals. But surprisingly, the petals of these mutants were constituted of only 60% of the cells of the WT. These cells were larger and had a higher ploidy [16]. Moreover, the constitutive expression of *CycD3* in *Arabidopsis* resulted in the induction and maintenance of cell division [17]. In tomato, three genes encoding CYCD3 were identified [22]. In our condition, the expression of *SlCycD3;1* and *SlCycD3;3* genes was stimulated by the exposure to BL, corresponding with the inhibition of endoreduplication. Whereas *SlCycD3;1* was expressed to the same extent in the WT and in the *7B-1* mutant, *SlCycD3;3* was less stimulated in the mutant than in the WT. These results indicate that these two genes are differentially regulated by the light, with *SlCycD3;3* being probably more specifically regulated by the *7B-1* mutation. We evidenced that the control of endoreduplication by blue light, via CYCD3, is part of the mechanisms leading to photomorphogenesis in tomato.

Cytokinins (CKs) mediate a number of light-regulated processes, notably photomorphogenesis. The relationship between light and CKs was suggested by Chory and coworkers [9]. Indeed, exogenously applied CKs, and particularly iP, induced several characteristics of the photomorphogenesis in dark-grown *Arabidopsis* seedlings, such as inhibition of hypocotyl growth or opening and greening of cotyledons [9]. Moreover, the characterization of mutants accumulating CKs or transgenic plants overproducing CKs established stronger evidence that CKs play an important role during photomorphogenesis [8], [33]. In our study, we investigated the role of endogenous CK during photomorphogenesis in tomato. For this purpose, endogenous CKs were quantified from both genotypes grown either in the dark or in continuous blue light. First, we identified that BL-grown seedlings of tomato accumulated higher content of free-base CKs than dark-grown seedlings. Second, we demonstrated that the three classes of CKs (*c*Z, *t*Z and iP) are accumulated in the hypocotyl of tomato. Because the content of *c*Z was similar in the hypocotyls of D- and BL-grown seedlings of both genotypes and remained very low in comparison to the two other CKs, we hypothesized that *c*Z does not play an essential role in photomorphogenesis. The relevant role of *c*Z in different physiological processes was already questioned in *Arabidopsis*. Indeed, mutants affected in *c*Z biosynthesis did not show a drastic morphological modification [34]. The *t*Z and derivatives accumulated to the same extent in both genotypes and accounted for the highest proportion of total CKs in the hypocotyl of D-grown tomato (up to 70%). This result is contrary to the data obtained by Gajdošová and coworkers who identified *c*Z as the most abundant CK in the leaves of mature tomato plants [35]. The discrepancy between the data reflects a hypothetical differential distribution of the various CKs within plant organs. This hypothesis is supported by the fact that *IPT* genes, encodings proteins catalyzing the first step of CK biosynthesis are spatially regulated [36]. The exposure to BL did not significantly affect the *t*Z content in the hypocotyl of both genotypes. The results obtained for iP and derivatives were the most interesting. Indeed, whereas iP content was very low in the hypocotyl of D-grown seedlings and identical in the two genotypes, the exposure to BL induced a strong accumulation of iP in the hypocotyl of cv. Rutgers (almost 6 times more) but not in the *7B-1* mutant which is either defective either in BL perception or BL signaling pathway [19]–[21]. The lack of the accumulation of endogenous iP in the mutant could be overcome by exogenous iP. Thus it appeared that exogenous iP induced a dose-dependent inhibition of the hypocotyl growth of the mutant grown in continuous blue light, whereas *t*Z and *c*Z were not efficient. We assumed that first the iP accumulation is specific from the response to blue light and second iP plays an important role in the BL-induced photomorphogenesis in tomato.

The homeostasis of CKs is a complex phenomenon based on synthesis, import rate, association and dissociation from conjugates (especially glucosides), export and catabolism [25]–[26]. The initial step of iP synthesis is catalyzed by the adenosine phosphate-isopentenyltransferase (IPT) which uses DMAPP and ATP or ADP to give an iP nucleotide, such as iP riboside 5′-triphosphate or iP riboside 5′-diphosphate, respectively. For a long time it was believed that to become active iP-nucleotides are converted to nucleobases by dephosphorylation and deribosylation, but genes encoding the corresponding enzymes catalyzing these reactions have not yet been identified. Very recently, the isolation and characterization of the cytokinin nucleoside 5′-monophosphate phosphoribohydrolases or LOG identified a novel direct pathway of free active iP from the iP riboside 5′-monophosphate [37]. In tomato, blue light induced the accumulation of the iP5′ MP forms but not of the iPR, indicating that at least during BL-induced morphogenesis, free iP is synthesized through the direct pathway mediated by LOG. This hypothesis was verified by the analysis of gene expression by qRT-PCR. Four *LOG* genes were identified from the tomato database and all of them belonged to the clade I previously described [24]. During BL-induced photomorphogenesis, *SlLOG6* and *SlLOG2* were strongly accumulated in the hypocotyl of cv. Rutgers but not in the mutant, consistent with the fact that the mutant is affected in the synthesis of free iP and that the mutation affects the regulation of *LOG* gene transcription. The analysis of the expression of up-stream genes, such as genes encoding IPT, could bring more insights concerning the full cascade leading to iP synthesis. Because the *SlLOG2* gene was the most abundant, we hypothesized that the corresponding protein could mediate the specific accumulation of iP. Therefore, further experiments are currently ongoing in order to confirm whether the over-expression of *SlLOG2 in planta* could trigger the increase in iP content and if the increase in iP content might induce a light-like phenotype in D-grown tomato seedlings. Beside the synthesis, the endogenous iP status was found to be regulated both by the association to glucosides and the inhibition of the degradation by the enzymes encoded by the *CKX* genes. Indeed, we could observe that the blue light induced an accumulation of the 9-O-glucoside form of iP in both genotypes, but to a greater extent in the *7B-1* mutant. In the meantime, we could observe that the exposure to BL inhibited the expression of the *SlCKX* genes, indicative of an inhibition of the CK degradation during photomorphogenesis. Because no difference was detected between the WT and the *7B-1* mutant it can be assumed that the regulation of *SlCKX* gene transcription is independent from the function encoded by the *7B-1* mutation.

CK signal transduction is mediated by a two-component system in which the signal is transmitted from a sensory histidine kinase to a response regulator. The histidine kinases AHK2, AHK3 and AHK4/CRE1/WOL act as CK receptors [38]–[40]. Riefler and coworkers demonstrated that AHK3 in combination with either AHK2 or CRE1/AHK4 mediated the photomorphogenesis triggered by exogenous CKs in dark-grown *Arabidopsis* seedlings [41]. LRG-911, an inhibitor of CK receptors [42], was used to investigate the involvement of the CK transduction pathway during the BL-induced photomorphogenesis in tomato. The inhibition of CK receptor by the addition of LRG-911 in the culture medium resulted in the stimulation of the growth of both cv. Rutgers and the *7B-1* mutant when grown in continuous BL, suggesting that the two-component system involving AHK3 or CRE1/AHK4 is involved in the BL-induced photomorphogenesis.

Since it was demonstrated that exogenous CKs induced photomorphogenesis in the D-grown seedlings [9], the question concerning the interaction between light and CKs during plant growth and development was raised. Thus it was assumed that common transcriptional targets could be regulated both by CKs and light [10], [43]–[45]. The transcription factor HY5 was identified as one of these regulators common to both CKs and light [10]. In our study, we did not clearly identify an interaction between CK and BL signaling pathway. Nevertheless, the use of the *7B-1* mutant defective in several responses to BL [19]–[21] provided more evidence on the interaction between CKs and light.

In this study, we identified that both accumulation of CKs – more specifically iP – and inhibition of endoreduplication are part of the mechanisms occurring during BL-induced photomorphogenesis. The relationship between CKs and endoreduplication is poorly documented. The relationship between CKs and endoreduplication in the control of growth was first reported by Riou-Khamlichi and coworkers [17]. These authors demonstrated that the expression of *CycD3* genes was not only higher in *Arabidopsis* mutants with a high content of CKs but was also stimulated by the application of exogenous CKs. They concluded that CKs activate *Arabidopsis* cell division through the induction of CYCD3 at the G1-S cell cycle phase transition. More recently, Dewitte and co-workers reported the importance of CYCD3 in promoting mitotic cycles and restraining endocycles, leading to the establishment of the normal cell number in shoot lateral organs. They also stated how CYCD3 are essential for CK-mediated responses [16].

**Figure 16:**
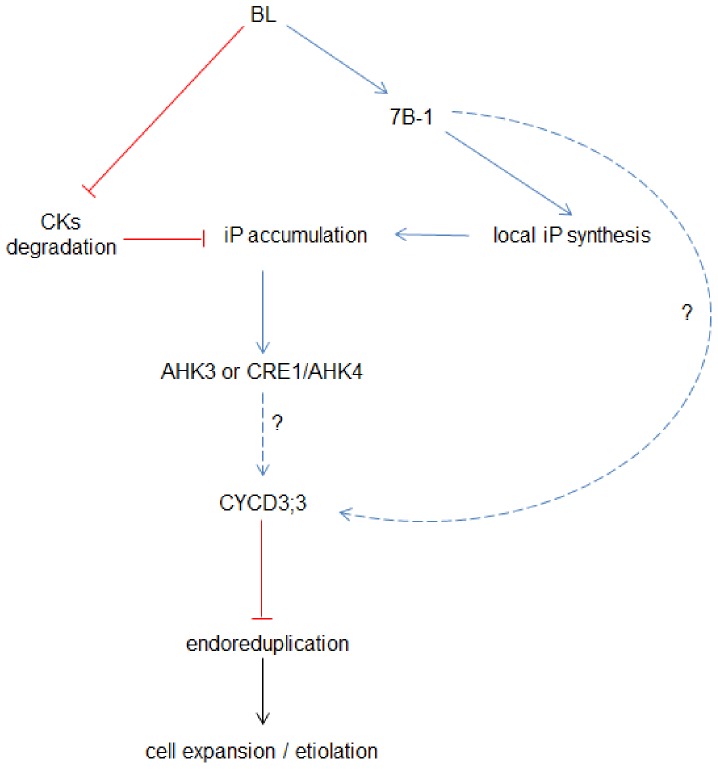
Proposed model illustrating how BL influences CKs to regulate the de-etiolation of tomato hypocotyls by the mean of the functional *7B-1* gene (blue lines indicate stimulatory effect, whereas red lines represent inhibitory effect). In this model, BL stimulates iP synthesis and inhibits CK degradation, leading to an overall accumulation of iP. iP stimulates the expression of *CycD3* genes, important regulators of the cell cycle and endocycle. A higher expression of *CycD3* genes inhibits the endoreduplication and allows the entry into cell differentiation.

Until now, our study identified how CKs and endoreduplication are differentially regulated during skotomorphogenesis and photomorphogenesis of tomato. Our attention came naturally to focus on the switch between these two developmental programs, referred as de-etiolation. As already mentioned, in tomato, like in *Arabidopsis*, during skotomorphogenesis, the epidermal cells of the hypocotyl expended across an acropetal gradient along the hypocotyl leading to a zone of active expansion just below the cotyledons. When the seedling emerges from the soil and senses the light, the growth rate of the expansion zone is partially inhibited. By the mean of CCD camera-capture imaging, we were able to follow the changes in the growth rate of the expending zone during de-etiolation. Like in *Arabidopsis* [28], when an etiolated seedling of tomato is exposed to BL, the inhibition of the fast growth rate, characteristic of the skotomorphogenesis, occurred in two phases: the first early phase, characterized by a rapid and drastic reduction in the growth rate (within 30–35 min), and the later second phase, characterized by a transient recovery of the growth rate until the establishment of a steady-state growth rate. The CK content, as well as the expression pattern of different genes encoding proteins involved in the synthesis and catabolism of CKs, was analyzed during the de-etiolation specifically in the expanding zone of hypocotyl just below the cotyldedons. The only interesting and significant result was the increase in iP and iP-precursors. The increase in free iP and precursors was correlated with the stimulation of *SlLOG6* and *SlLOG2* gene transcription and a concomitant inhibition of the transcription of the *SlCKX5* gene. The use of promoter-reporter gene fusions in transgenic *Arabidopsis* lines demonstrated that CK can be locally synthesized, act as a local signal and can be catabolized at distant sites [37]. The compartmentalization of the different CKs within plant organs with iP accumulating in phloem sap and acting as a basipetal or a systemic signal in the aerial part of the plants was also reported [37]. These information taken all together allow us to hypothesize that upon exposure to BL a local synthesis of iP, together with an inhibition of its degradation, occurred in the expending zone of the hypocotyl, leading to the inhibition of the fast growth rate characteristic from the skotomorphogenesis. Concomittantly, the cell expansion is limited by the inhibition of the endoreduplication, probably mediated by the mean of the CYCD3;3 protein as it might be hypothesized from the data of analysis of gene expression. Because, both iP accumulation and inhibition of endoreduplication are significantly affected by the exposure to BL only after 1 h of illumination, we assumed that these two responses are not involved in the first rapid phase of de-etiolation but more surely participate in the establishment of the later steady-state growth of rate. Finally the same conclusions could be made for the BL-mediated de-etiolation and for the BL-mediated photomorphogenesis, leading us to the conclusion that several mechanisms are intiated during the switch from skotomorphogenesis to photomorphogenesis, i.e. during de-etiolation and that these mechanisms are maintained during the photomorphogenesis. In conclusion, we propose a model to understand the BL-mediated photomorphogenesis in tomato seedlings (Figure 16). In this model, the accumulation of iP under BL condition results both from the stimulation of the synthetic pathway and the inhibition of the catabolic pathway; iP, through the two-component system, stimulates the expression of *CycD3* genes that mediates the inhibition of endoreduplication and allows the entry into the differentiation.

## Materials and Methods

### Plant Material and Growth Conditions

The tomato *Solanum lycopersicum* L. cv. Rutgers was primarily used in this study. For some limited experiments, the spontaneous single-gene recessive *7B-1* mutant, affected in its response to BL, was also analyzed [18]–[21]. Sterile cultures were obtained as described in [21]. Seedlings were grown at 23°C in culture chambers equipped with fluorescent lamps providing blue light (BL; TL-D 36W/Blue, Phillips; total photon fluence rate 10 µmol.m^−2^.s^−1^). The light spectrum was measured using a portable spectroradiometer (model LI-1800; Li-COR, Lincoln, NE). For the dark conditions, Petri dishes were wrapped in aluminum foil, and placed in the same culture chamber under the same temperature regime.

### Hypocotyl Growth and Cell Length Measurement

Germinated seeds were transferred to a new medium and Petri dishes were placed vertically in the culture chamber at 23°C. Seedlings grew for 5 days under continuous BL or in the dark (D). Hypocotyl length was measured with a ruler. Results represent averages of 10 seedlings ± SE.

For the cell length measurement, the middle region of the hypocotyl of 5 days-old seedlings was excised and fixed for 30 min in 96% ethanol. Samples were immerged for 20 min in different gradient of ethanol solution (80%, 70% and 50%), washed 2 times in water for 10 min and finally stained for 10 min in a solution of toluidine blue (1%, w/v). Fragments were mounted in a drop of water and pictures were taken with a microscope (Olympus BX60, Japan) equipped with a camera (Olympus DP71, Japan). Cell length was measured using the ImageJ software (a public domain, Java-based image processing program developed at the National Institutes of Health; http://imagej.nih.gov/ij/download.html). For each genotype and light conditions, cells of 5 hypocotyls were monitored.

### Ploidy Determination

Epidermis was peeled from hypocotyls of the two genotypes grown for 5 days in continuous BL (10 µmol.m^−2^.s^−1^). Nuclei were extracted in LB01 buffer (15 mM TrisHCl pH 7.5, 2 mM Na_2_EDTA, 0.5 mM spermine, 80 mM KCl, 20 mM NaCl, 15 mM β-mercaptoethanol and 0.1% Triton X100 (v/v) [46] and stained with propidium iodide to a final concentration of 26 µg/ml. Analysis was performed with a flow cytometer CyFlow ML (Partec). For each sample, 3000 nuclei were measured. The gain of the instrument was adjusted so that the G1 peak of standard corresponded to the channel 100. The ploidy level of other peaks was determined compared to the position of the G1 peak. The ratio between the G1 (2C) phase/G2 (4C) phase was 1.90 (averaged index); the ratio between the G1 (2C) phase/G3 (8C) phase was 3.81 (averaged index). Analyses of the samples were obtained from histograms with a coefficient of variance between 4 and 7. For one sample, epidermis of 3 to 5 seedlings was pooled. *Solanum lycopersicum* cv. Stupicke was used as a standard with 2C = 1.96 pg DNA [47].

### Extraction and Quantification of Endogenous Cytokinins

Extraction, purification, and quantification of endogenous cytokinins from the entire hypocotyl was performed by ultra-performance liquid chromatography–electrospray tandem mass spectrometry according to the method previously described [48]–[49]. Germinated seeds from both genotypes were transferred on medium, placed vertically in a culture chamber and grown for 5 days in the D or in continuous BL (10 µmol.m^−2^.s^−1^). Results represent the average ± SE of 4 independent biological repeats.

### Exogenous Cytokinins and Inhibitors of CK Receptors

Germinated seeds of both genotypes were transferred to MS medium and placed in the D for 2 days in order to induce the etiolation of the seedlings. Then the etiolated seedlings were transferred on medium containing different concentration of CKs (iP, *t*Z or *c*Z) or 5 µM of LRG-991, an inhibitor of CK receptors. Both Petri dishes containing CKs and the inhibitor were placed in continuous BL. After 24 h, the length of the hypocotyl was measured with a ruler to the nearest millimeter. The results represent the average ± SE. The number of seedlings measured is indicated in the legend of the specific figure. The iP, *t*Z and *c*Z were kindly provided by P. Galuszka (Department of Biochemistry, Palacky University, Olomouc, Czech Republic); LRG-991 was kindly provided by L. Spíchal (Laboratory of Growth Regulators, Palacky University, Olomouc, Czech Republic).

### Analysis of Gene Expression by Quantitative RT-PCR after 24 h of Exposure to BL in the cv. Rutgers and the *7B-1* Mutant

Germinated seeds of both genotypes transferred on new medium were placed vertically in a culture chamber at 23°C in the D for two additional days. The upper part of the hypocotyls was harvested immediately (0 min BL) or 24 h following exposure to BL. Total RNA were extracted using an RNeasy Plant Mini Kit (QIAGEN), followed by an additional DNaseI treatment (Takara) for 30 min at 37°C. DNAseI was heat-inactivated for 10 min at 65°C and RNA were purified by a phenol:chloroform:iso-amylalcohol (25∶24∶1) step. The reverse transcription was done from 2 µg of total RNA in a reaction containing 1X RT buffer, 0.5 mM dNTP, 2.5 µM oligo(dT)_20_ primer, 20 u of RNAse inhibitor (Takara) and 200 u of PrimeScript™ RTase (Takara). The reaction mixture was incubated at 42°C for 60 min followed by heat inactivation at 70°C for 15 min. RNA were digested by 5 u of RNaseH (Takara) for 20 min at 37°C. The cDNA were subsequently purified on a column and eluted in sterile RNAse-free water. For quantitative real-time PCR, cDNA samples were used in a reaction containing SYBR Premix ExTaq (Takara) PCR Master Mix and 200 nM of each primer. Two technical repeats were run for each sample on the Mx3000P thermo cycler (Stratagene) in a two-steps amplification program. The initial denaturation at 94°C for 10 sec was followed by 40 cycles: 94°C for 5 sec, 60°C for 20 sec. A dissociation curve was obtained for each sample. Three independent biological repeats were analyzed for each sample. Cycle threshold values were normalized in respect to *EF1*α gene, and the efficiency of amplification. Differences in the cycle numbers during the linear amplification phase between the samples and the ΔΔC_T_ method were used to determine the fold change in the gene expression. The results are expressed in term of “fold change”. The relative quantification was made compared to the sample “cv. Rutgers-D”. Sequences of primers are given in table 2, primers for tomato CKX were kindly provided by H. Pospíšilová (Department of Molecular Biology, Centre of the Region Haná for Biotechnological and Agricultural Research, Olomouc, Czech Republic).

**Table 2:** Sequences of primer combinations used in this study to investigate the expression of different genes during BL-induced de-etiolation in tomato.

gene	accession number	sequences of primer	
*SlCYCD3;1*	AJ002588	F	5′-TCTTGAGCTAATGGACACTGC
		R	5′-ATCGAAGATGCTACTGACCATG
*SlCYCD3;2*	AJ002589	F	5′-TTTGTTTTGGGAAGATGAAGAGC
		R	5′-TGAGCATTCACTTTAAGAATCCATTC
*SlCYCD3;3*	AJ002590	F	5′-CAAGGAGAAGGTGGAAGGATG
		R	5′-CTGTTGGACTCCCTGGTAATG
* SlLOG1*	SGN-U579950	F	5′-AGGATTGCTGAATGTGGATGG
		R	5′-GACTAGCTCCTTTGACGATGG
* SlLOG2*	SGN-U585768	F	5′-TGTTGGAGAAGTAAGAGCAGTG
		R	5′-ATGAATGCCTAGTTGAGCCC
* SlLOG4*	SGN-U591591	F	5′-CCAAGACACTCATGCCTAGAG
		R	5′-TCCTCAAGTGTGCCATAACC
* SlLOG6*	SGN-U582698	F	5′-TGGGAGAGGTTAAAGCAGTTG
		R	5′-AATTCCGAGTTGAGACCACG
* SlCKX1*	SGN-U342971	F	5′-TCATGCATCAGAGCTAATACTTCGA
		R	5′- CTAGAAGATTGAGCCATGGATGTG
* SlCKX5*	SGN-U223622	F	5′-GGGTTCACAAGGCCGAGTTA
		R	5′-GCCATGGATGTGGCACTTC
* EF1α*	X14449	F	5′- CCCAAGAGGCCATCAGACAA
		R	5′-CAACAGGGACAGTTCCAATACCA

### Determination of the Elongating Zone

Under green safety light, marks were made each 1 cm along the hypocotyl of two day-old etiolated seedlings. Seedlings were quickly scanned and immediately returned either to the dark or in continuous BL for 24 h. Seedlings were again scanned and the length of the different zones could be evaluated using ImageJ software. The results represent the ratio of the length of the corresponding zone after 24 h of treatment and the initial length. For each condition, 8 seedlings were followed.

### Camera-assisted Imaging

The DISP (Digital Images Sequence Processing) method was used to characterized the de-etiolation of 2 day-old etiolated seedlings upon exposure to BL. Three etiolated seedlings on the surface of MS medium in dish, vertically positioned in a culture chamber, were monitored each time. Hypocotyl images were acquired with high resolution, monochrome CCD cameras (Scorpion SCOR-20SO; Point Grey Research, Vancouver, BC, Canada), positioned in front of the dish and equipped with a standard objective lens (25 mm; Cosmicar/Pentax, The Imaging Source, Bremen, Germany) and an infrared interference filter (880 nm; Edmund Optics, Karlsruhe, Germany). Seedlings were placed in the dark for 60 min then the BL was switched on (TL-D 36W/Blue, Phillips; total photon fluence rate 10 µmol.m^−^^2^.s^−1^). Grey value images were taken every minute for 2 additional hours and saved in a multi-tiff format. Image sequences were evaluated and the area relative growth rates were calculated as described in [50]. Eleven seedlings were measured and the data represent the average ± SE.

### Statistical Analysis

The statistical significance of the treatment differences was assessed by the one- or two-way ANOVA followed by the Bonferroni *post*-*hoc* test or by the *t*-test of Student performed with the Statistica software version 6 (Stat Soft, Inc.). The tool allowing the description of correlation was also used.

## Supporting Information

Figure S1 Expression by qRT-PCR of *SlCYCD3;1* (A) and *SlCYCD3;3* (B) in 2-do etiolated seedlings of cv. Rutgers and *7B-1* mutant subjected for 24 additional hours to the D or BL.
(TIF)

Figure S2 Expression by qRT-PCR of *SlLOG1* (A), *SlLOG2* (B), *SlLOG4* (C) and *SlLOG6* (D) in 2-do etiolated seedlings of cv. Rutgers and *7B-1* mutant subjected for 24 h to the D or BL.
(TIF)

Figure S3 Expression by qRT-PCR of *SlCKX1* (A) and *SlCKX5* (B) in 2-do etiolated seedlings of cv. Rutgers and *7B-1* mutant subjected for 24 h to the D or BL.
(TIF)
