# Supplementary figures and images for: Effect of Blue Light on Endogenous Isopentenyladenine and Endoreduplication during Photomorphogenesis and De-Etiolation of Tomato (Solanum lycopersicum L.) Seedlings
Source: PLoS One. 2012 Sep 25;7(9):e45255. doi: 10.1371/journal.pone.0045255 (PMC3458014; doi:10.1371/journal.pone.0045255)

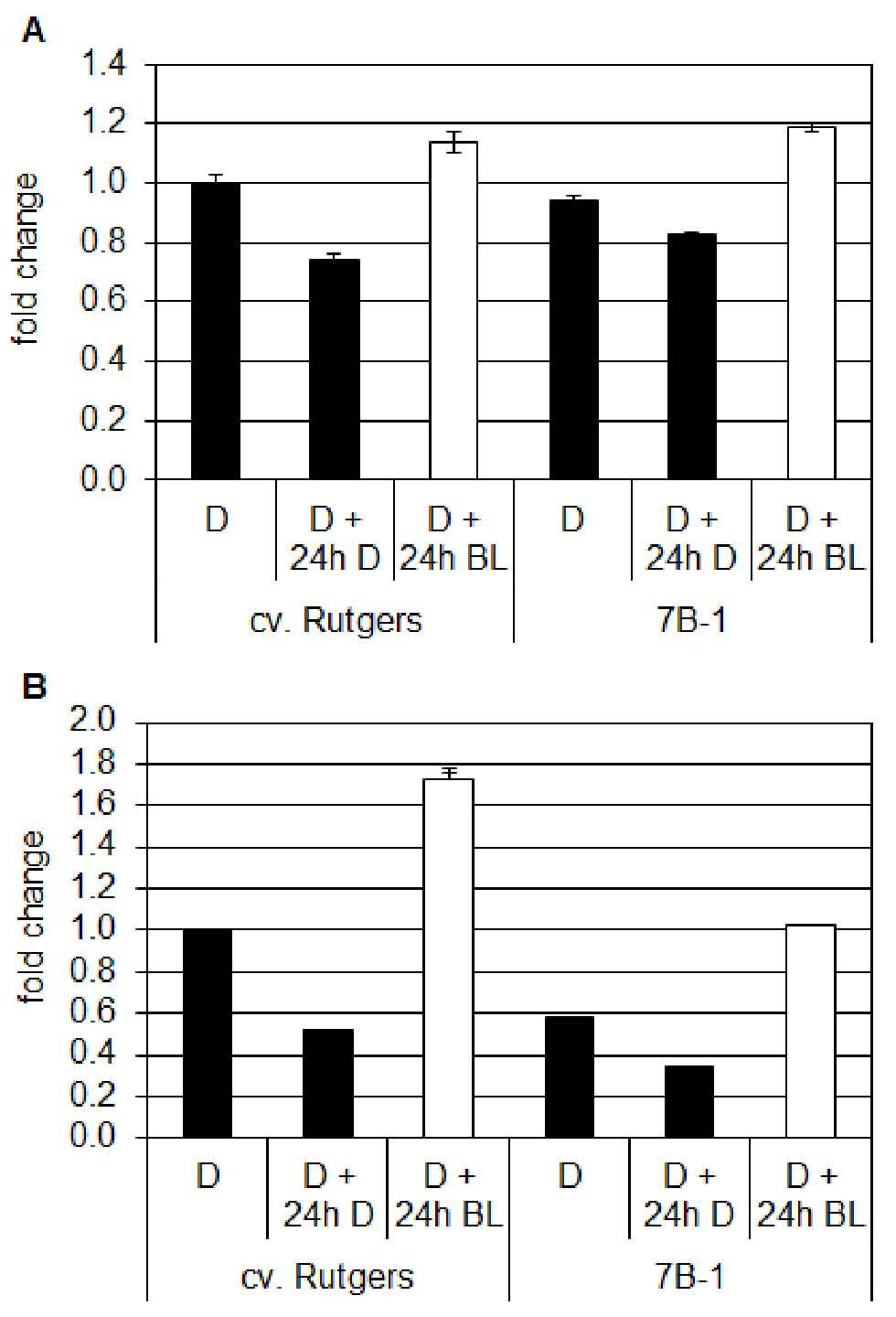

Supplement: Figure S1 — Expression by qRT-PCR of SlCYCD3;1 (A) and SlCYCD3;3 (B) in 2-do etiolated seedlings of cv. Rutgers and 7B-1 mutant subjected for 24 additional hours to the D or BL. (TIF) [file pone.0045255.s001.tif]

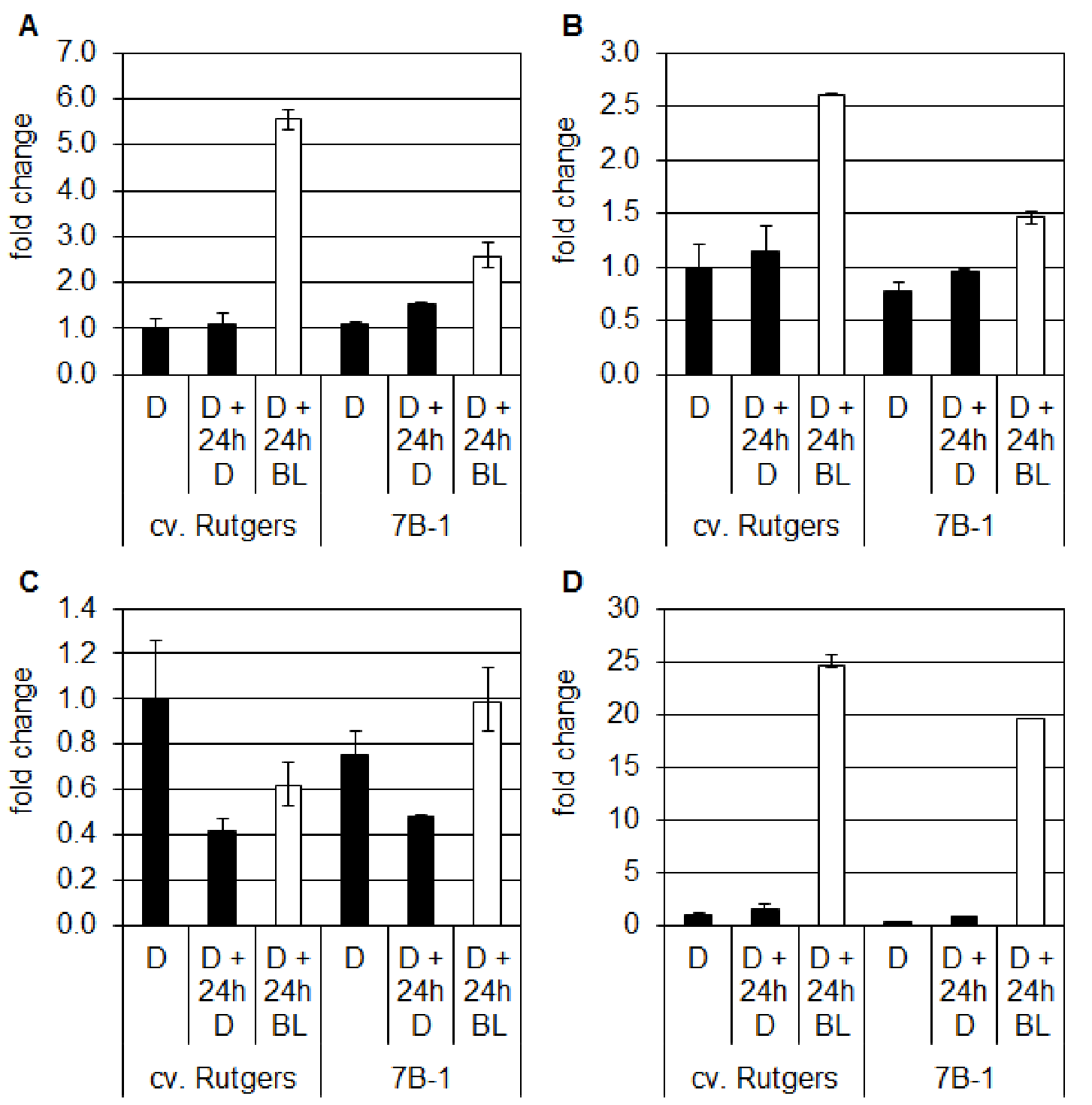

Supplement: Figure S2 — Expression by qRT-PCR of SlLOG1 (A), SlLOG2 (B), SlLOG4 (C) and SlLOG6 (D) in 2-do etiolated seedlings of cv. Rutgers and 7B-1 mutant subjected for 24 h to the D or BL. (TIF) [file pone.0045255.s002.tif]

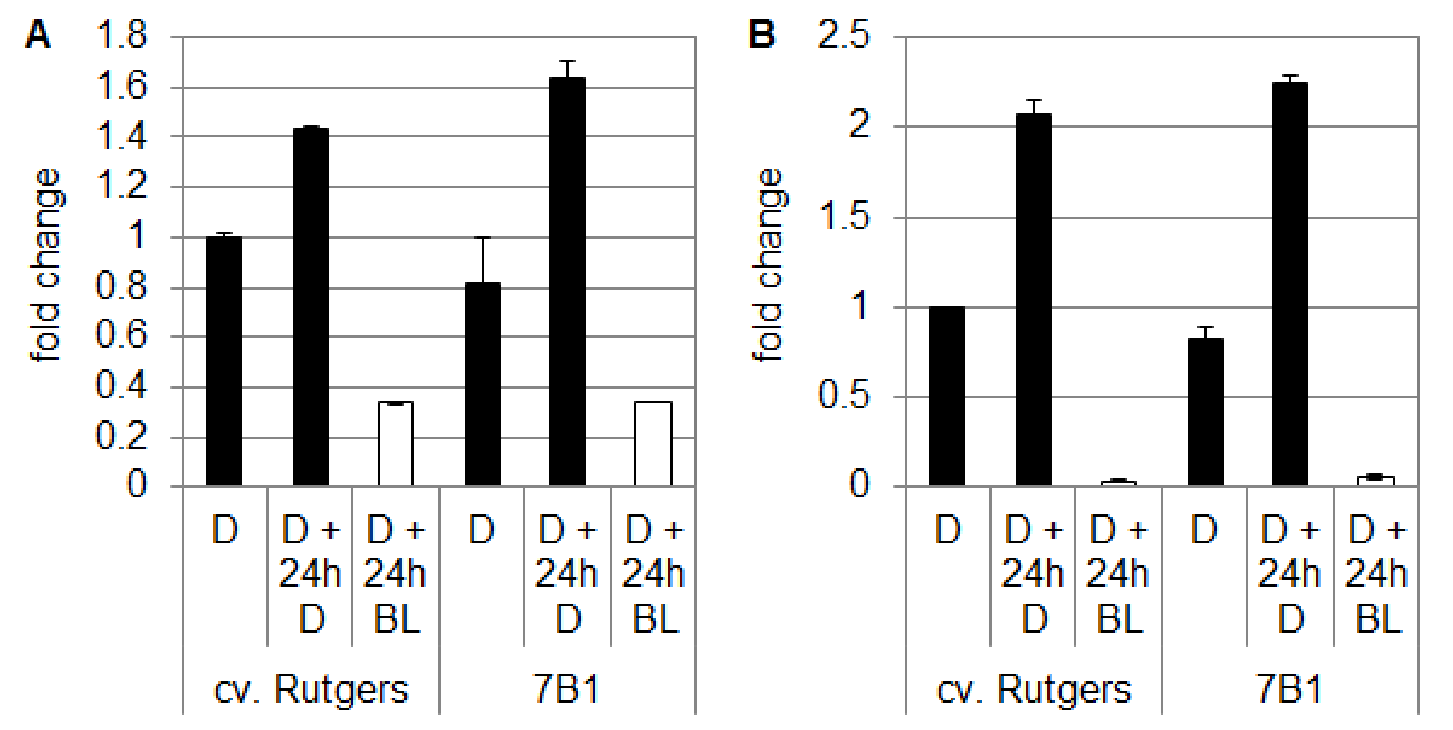

Supplement: Figure S3 — Expression by qRT-PCR of SlCKX1 (A) and SlCKX5 (B) in 2-do etiolated seedlings of cv. Rutgers and 7B-1 mutant subjected for 24 h to the D or BL. (TIF) [file pone.0045255.s003.tif]
